# Breastfeeding, Prepubertal Adiposity, and Development of Precocious Puberty

**DOI:** 10.1001/jamanetworkopen.2025.27455

**Published:** 2025-08-18

**Authors:** Yunsoo Choe, Soorack Ryu, Jinjoo Choi, Jae Yoon Na, Kyung Suk Lee, Yong Joo Kim, Seung Yang

**Affiliations:** 1 Department of Pediatrics, Hanyang University Guri Hospital, Guri, Republic of Korea; 2 Department of Pediatrics, Hanyang University College of Medicine, Seoul, Republic of Korea; 3 Biostatistical Consulting and Research Laboratory, Medical Research Collaborating Center, Hanyang University, Seoul, Republic of Korea; 4 Department of Medicine, Hanyang University College of Medicine, Seoul, Republic of Korea; 5 Department of Pediatrics, Hanyang University Medical Center, Seoul, Republic of Korea

## Abstract

**Importance:**

The incidence of central precocious puberty (CPP) is increasing globally, raising concern about its potential long-term health consequences. However, the association between early-life feeding practices and CPP is poorly understood.

**Objective:**

To investigate the association of breastfeeding during the first 4 to 6 months of life with CPP in boys and girls and whether this association is mediated by prepubertal adiposity.

**Design, Setting, and Participants:**

This nationwide, retrospective cohort study used health claims data from the South Korean National Health Insurance Service Database between January 1, 2007, and December 31, 2020. Children who underwent routine health checkups at 4 to 6 months (examination 1) and 66 to 71 months (examination 7) were eligible. Children with comorbidities, who died during the follow-up period, who had missing information, or who were diagnosed with CPP before age 6 years were excluded. Data were analyzed between October 9, 2024, and January 14, 2025.

**Exposure:**

Feeding practice patterns collected through a primary caregiver–reported questionnaire during examination 1.

**Main Outcomes and Measures:**

The primary outcome was the incidence of CPP, defined by *International Statistical Classification of Diseases, Tenth Revision* diagnostic codes and administration of gonadotropin-releasing hormone agonists. The association between feeding practices and incidence of CPP was assessed using a multivariable Cox proportional hazards model to estimate adjusted hazard ratios (AHRs) and 95% CIs. Causal mediation analysis within a counterfactual framework was conducted to quantify mediation by childhood overweight or obesity.

**Results:**

Among 322 731 children (58.1% girls), 46.0% were exclusively breastfed, 34.9% were formula-fed, and 19.1% were mixed-fed. Compared with exclusively breastfed children, formula-fed children had the greatest risk of CPP in boys (AHR, 1.16; 95% CI, 1.10-1.21) and girls (AHR, 1.60; 95% CI, 1.24-2.06), followed by mixed-fed boys (AHR, 1.14; 95% CI, 1.07-1.20) and girls (AHR, 1.45; 95% CI, 1.07-1.97). Mediation through prepubertal adiposity accounted for 7.2% (bootstrap 95% CI, 4.5%-12.1%) and 17.8% (bootstrap 95% CI, 6.6%-30.0%) of the association between formula feeding and CPP in boys and girls, respectively.

**Conclusions and Relevance:**

In this nationwide, retrospective cohort study, breastfeeding during the first 4 to 6 months of life was associated with a lower risk of CPP in boys and girls. This association was partially mediated by prepubertal adiposity, highlighting the critical role of early-life nutrition in pubertal timing.

## Key Points

**Question:**

Is exclusive breastfeeding in early infancy associated with central precocious puberty?

**Findings:**

In this cohort study of 322 731 children, those who were exclusively breastfed had a lower risk of central precocious puberty than those who were formula- or mixed-fed. This association was mediated by prepubertal adiposity.

**Meaning:**

These findings suggest that feeding practices in early life are associated with pubertal timing both directly and through childhood adiposity.

## Teaser

This cohort study investigates the association of breastfeeding during the first 4 to 6 months of life with central precocious puberty in Korean boys and girls and whether the association is mediated by prepubertal adiposity.

## Introduction

The age at pubertal onset has progressively declined over recent decades,^1^ accompanied by a rising incidence of central precocious puberty (CPP) worldwide.^2,3^ Precocious puberty is associated with an increased risk of adult health problems, such as diabetes, cardiovascular disease, and cancer.^4^ While environmental factors such as soy consumption^5^ and exposure to endocrine-disrupting chemicals^6^ have been identified as potential risk factors for CPP, childhood obesity is recognized as a major predisposing factor.^7^

Human milk is the first source of nutrition that newborns receive immediately after birth and usually serves as the sole source of nutrition during the first 4 to 6 months of life before complementary foods are introduced. The World Health Organization recommends exclusive breastfeeding for the first 6 months of life and continued breastfeeding with complementary foods for up to age 2 years or older.^8^ Breastfeeding benefits neurodevelopment and provides long-term protection against various diseases, including obesity, diabetes, and cancer.^9,10,11,12^ Among the various environmental factors that influence the risk of subsequent obesity, such as familial lifestyle and parental obesity, early feeding practices play a critical role in metabolic programming during infancy.^13^

The effects of prepubertal obesity on pubertal development are thought to differ between boys and girls.^14,15^ Several studies investigating the association between breastfeeding and pubertal development have shown different directions between sexes.^16,17^ The mechanisms underlying the differences in adiposity-related pubertal development remain unclear, and research on the association of breastfeeding practices with pubertal onset in boys is scarce.

This study investigated the association between primary feeding type during the first 4 to 6 months of life and the risk of CPP in South Korea. Additionally, we explored whether being overweight or obese during the prepubertal period mediates this association.

## Methods

### Data Source

This retrospective cohort study used data from the National Health Information Database (NHID) and National Health Screening Program for Infants and Children (NHSPIC), both established by the National Health Insurance Service (NHIS) in South Korea. This study was approved by the Institutional Review Board of Hanyang University Guri Hospital with a waiver of informed consent because the study used deidentified secondary data from the NHIS, and no personally identifiable information was accessible to the researchers. This study adhered to the Strengthening the Reporting of Observational Studies in Epidemiology (STROBE) reporting guideline.

The NHIS is a mandatory, universal health insurance program covering more than 98% of the South Korean population, and the NHID is a comprehensive public database that includes data on health care use; sociodemographic variables; diagnosis codes based on the *International Statistical Classification of Diseases, Tenth Revision* (*ICD-10*); prescription records; and mortality.^18^ The NHSPIC is a standardized health checkup program for preschool-aged children (4-71 months), including anthropometric measurements and developmental assessments through caregiver-reported questionnaires.^19^ It comprises 7 scheduled examinations at age 4 to 6 months, 9 to 12 months, 18 to 24 months, 30 to 36 months, 42 to 48 months, 54 to 60 months, and 66 to 71 months. We included children who underwent both examination 1 (4-6 months) and examination 7 (66-71 months) to evaluate primary feeding practices and prepubertal weight status.

### Study Population

We screened children born between January 1, 2007, and December 31, 2010. Children who completed both examinations 1 and 7 were eligible. We excluded children with comorbidities, who died during the follow-up period, who were missing information, or who had a diagnosis of CPP before age 6 years. The final sample was followed up using data available through December 31, 2020, but follow-up for each child was limited to age 10 years for boys and age 9 years for girls, depending on their birth year.

### Risk Factors and Covariates

Baseline demographic characteristics were obtained from the NHID. Socioeconomic status was categorized into quartiles based on health insurance premiums to reflect household income. Residence was classified as urban or rural based on the registered address as of January 1 of the birth year. Preterm births, multiple births, and cesarean deliveries were identified using *ICD-10* diagnostic codes. Birth weight and primary feeding type were collected from a primary caregiver–reported questionnaire during examination 1. Feeding type was categorized as exclusively breastfed, formula-fed, or mixed-fed. Anthropometric measurements from examination 7 were used to calculate the body mass index (BMI) (weight in kilograms divided by height in meters squared). Based on the 2007 Growth Chart for Korean Children,^20^ BMI percentiles for age and sex were used to classify obesity (≥95th percentile), overweight (85th-95th percentiles), and normal weight (<85th percentile). Overweight and obese individuals were grouped for analysis. Maternal variables included age at childbirth and prenatal comorbidities. The detailed definitions of the variables are provided in eTable 1 in Supplement 1.

Potential confounders were selected a priori based on clinical relevance and previous literature and refined using a directed acyclic graph approach^21^ (eFigure 1 in Supplement 1). Final covariates included preterm birth (yes, no), low birth weight (yes, no), multiple birth (yes, no), cesarean delivery (yes, no), maternal age at delivery (<25, 20-35, ≥35 years), gestational diabetes (yes, no), gestational hypertension (yes, no), socioeconomic status (first to fourth quartile), and residence (urban, rural). Prepubertal overweight or obesity was not included in the main analysis as it was considered a potential mediator.

### Definition of CPP

Children who developed breast budding before age 8 years in girls and testicular enlargement greater than 4 mL before age 9 years in boys, along with advanced bone age, were suspected of precocious puberty and underwent a gonadotropin-releasing hormone (GnRH) stimulation test. Central precocious puberty was diagnosed when the peak luteinizing hormone level exceeded 5.0 IU/L.^22^ Considering the average lag of approximately 1.5 years from the initial recognition of secondary sexual development to the diagnosis of CPP in hospitals,^23^ the NHIS in South Korea permits GnRH agonist treatment for CPP to begin before age 9 years for girls and age 10 years for boys and to continue until age 12 years for girls and age 13 years for boys. In our study, we defined CPP as follows^2,22^: (1) at least 1 outpatient or inpatient visit with a primary diagnosis corresponding to the *ICD-10* codes of E22.8, E30.1, or E30.8 and (2) at least 1 administration of a GnRH agonist (leuprolide acetate, triptorelin pamoate, or triptorelin acetate) before age 9 years for girls (up to age 8 years and 364 days) and age 10 years for boys (up to age 9 years and 364 days). The date of diagnosis was defined as the date of the first GnRH agonist administration.

### Statistical Analysis

The data analysis was performed between October 9, 2024, and January 14, 2025. Data are expressed as the mean and SD for continuous variables or the number and percentage for categorical variables. To compare the 3 groups based on feeding type (exclusively breastfed, formula-fed, and mixed-fed), we conducted analysis of variance and χ^2^ tests for continuous and categorical variables, respectively. A multivariable Cox proportional hazards model was performed to identify factors associated with CPP, and the results are presented as adjusted hazard ratios (AHRs) and 95% CIs. The proportional hazards assumption was evaluated and confirmed using Schoenfeld residuals. Kaplan-Meier curves were plotted to visualize CPP-free survival estimates according to primary feeding type. Sensitivity analyses were performed to confirm the robustness of the findings. We conducted a regression-based mediation analysis described by VanderWeele^24^ to examine whether prepubertal overweight or obesity mediates the association between primary feeding type and CPP. Statistical analyses were conducted using SAS Enterprise Guide, version 7.1 (SAS Institute Inc) and R, version 4.4.0 (R Foundation for Statistical Computing). A 2-sided *P* < .05 was considered statistically significant. Additional details are provided in the eMethods in Supplement 1.

## Results

### Baseline Characteristics of the Study Participants

We initially screened 1 877 734 children born between 2007 and 2010. Among them, 355 780 children who completed both examinations 1 and 7 were eligible (Figure). We excluded 33 049 children who had comorbidities, died during the follow-up period, had missing information, or were diagnosed with CPP before age 6 years. Among the 322 731 participants included in the study, 135 232 (41.9%) were boys and 187 499 (58.1%) were girls, with a mean (SD) birth weight of 3.19 (0.49) kg. During the first 4 to 6 months of life, 46.0% of children were exclusively breastfed, 34.9% were formula-fed, and 19.1% were mixed-fed (Table 1). Boys were more likely to be formula- and mixed-fed than exclusively breastfed (43.4% and 44.0% vs 39.9%; *P* < .001). Preterm birth, multiple birth, and cesarean delivery were more common among formula-fed children (3.1%, 5.6%, and 45.2%, respectively) compared with mixed-fed (2.3%, 4.4%, and 39.5%, respectively) and exclusively breastfed (1.2%, 1.6%, and 35.4%, respectively) children (*P* < .001 for all comparisons). Formula-fed children also had the lowest mean birth weight compared with mixed-fed and exclusively breastfed children (mean [SD], 3.15 [0.51] vs 3.19 [0.50] and 3.22 [0.46] kg, respectively; *P* < .001) and the highest proportion of low birth weight (<2.5 kg) (6.4% vs 4.9% and 3.0%, respectively; *P* < .001). The prevalence of overweight or obesity in NHSPIC examination 7 was similar in the formula-fed and mixed-fed groups but lower in the exclusively breastfed group (18.7% vs 18.7% vs 17.6%, respectively; *P* < .001), as was the prevalence of CPP (3.0% vs 3.0% vs 2.8%, respectively; *P* < .001). Significant differences were also observed in maternal age, the prevalence of gestational diabetes and hypertension, socioeconomic status, and residence across the feeding groups (Table 1).

**Figure.**
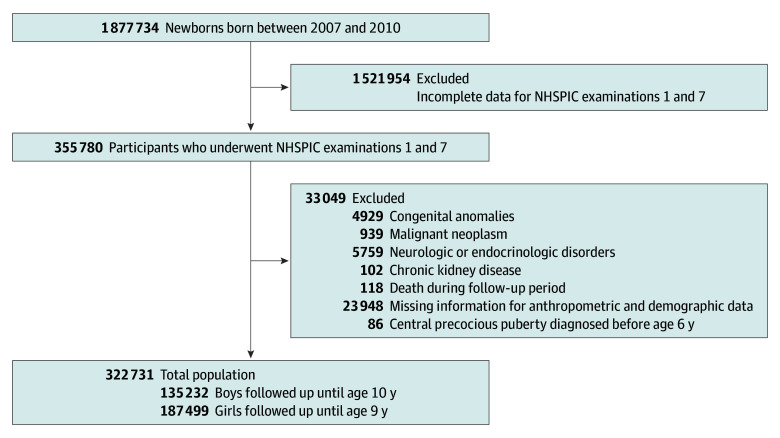
Flowchart of the Study Population The sum of participants excluded for each reason exceeds the total number of exclusions (n = 33 049) because some individuals met more than 1 exclusion criterion. NHSPIC indicates National Health Screening Program for Infants and Children in Korea.

**Table 1.** Baseline Characteristics of the Study Participants by Primary Feeding Type

Characteristic	Participants, No. (%)			*P* value
	Exclusively breastfed (n = 148 402)	Formula-fed (n = 112 738)	Mixed-fed (n = 61 591)	
**Child**				
Sex				
Female	89 208 (60.1)	63 776 (56.6)	34 515 (56.0)	<.001
Male	59 194 (39.9)	48 962 (43.4)	27 076 (44.0)	<.001
Preterm birth	1772 (1.2)	3471 (3.1)	1393 (2.3)	<.001
Birth weight, mean (SD), kg	3.22 (0.46)	3.15 (0.51)	3.19 (0.50)	<.001
Low birth weight (<2.5 kg)	4386 (3.0)	7228 (6.4)	3042 (4.9)	<.001
Multiple birth	2340 (1.6)	6308 (5.6)	2730 (4.4)	<.001
Cesarean delivery	52 566 (35.4)	50 982 (45.2)	24 350 (39.5)	<.001
Overweight or obesity^a^	26 162 (17.6)	21 074 (18.7)	11 544 (18.7)	<.001
Central precocious puberty	4126 (2.8)	3428 (3.0)	1820 (3.0)	<.001
**Maternal**				
Age at delivery, y				
<25	4208 (2.8)	3833 (3.4)	1703 (2.8)	<.001
25-35	124 792 (84.1)	89 614 (79.5)	49 337 (80.1)	
≥35	19 402 (13.1)	19 291 (17.1)	10 551 (17.1)	
Gestational diabetes	9298 (6.3)	8269 (7.3)	4255 (6.9)	<.001
Gestational hypertension	2214 (1.5)	2929 (2.6)	1169 (1.9)	<.001
Socioeconomic status, quartile				
First (lowest)	36 488 (24.6)	29 231 (25.9)	14 881 (24.2)	<.001
Second	36 809 (24.8)	28 786 (25.5)	15 150 (24.6)	
Third	37 570 (25.3)	27 491 (24.4)	15 638 (25.4)	
Fourth (highest)	37 535 (25.3)	27 230 (24.2)	15 922 (25.9)	
Residence				
Urban	66 475 (44.8)	49 031 (43.5)	28 538 (46.4)	<.001
Rural	81 927 (55.2)	63 707 (56.5)	33 008 (53.6)	

^a^ From the seventh infant health checkup (conducted at age 66-71 months).

### Associations Between Primary Feeding Type and CPP

The results of the Cox proportional hazards analyses for identifying the factors associated with CPP are presented in Table 2. After adjusting for potential risk factors, primary feeding type was significantly associated with CPP in both boys and girls. Specifically, compared with exclusively breastfed boys, formula-fed boys had the greatest risk of CPP (AHR, 1.16; 95% CI, 1.10-1.21; *P* < .001), followed by mixed-fed boys (AHR, 1.14; 95% CI, 1.07-1.20; *P* < .001). The association was more pronounced in girls. Compared with exclusively breastfed girls, the highest risk of CPP was observed in formula-fed girls (AHR, 1.60; 95% CI, 1.24-2.06; *P* < .001), followed by mixed-fed girls (AHR, 1.45; 95% CI, 1.07-1.97; *P* < .001). Kaplan-Meier survival curves showed significant differences in the cumulative incidence of CPP by primary feeding type in both sexes, with the lowest risk observed among exclusively breastfed children (log-rank *P* < .001) (eFigure 2 in Supplement 1).

**Table 2.** Associations Between Primary Feeding Type and Central Precocious Puberty in Children Stratified by Sex

Variable	Male (n = 135 232)		Female (n = 187 499)	
	AHR (95% CI)	*P* value	AHR (95% CI)	*P* value
Primary feeding type				
Exclusively breastfed	1 [Reference]	NA	1 [Reference]	NA
Formula-fed	1.16 (1.10-1.21)	<.001	1.60 (1.24-2.06)	<.001
Mixed-fed	1.14 (1.07-1.20)	<.001	1.45 (1.07-1.97)	<.001
Preterm birth				
No	1 [Reference]	NA	1 [Reference]	NA
Yes	1.42 (0.65-3.11)	.38	1.26 (1.06-1.50)	.009
Low birth weight				
No	1 [Reference]	NA	1 [Reference]	NA
Yes	1.99 (1.21-3.26)	.007	1.17 (1.05-1.30)	.003
Multiple birth				
No	1 [Reference]	NA	1 [Reference]	NA
Yes	1.10 (0.61-1.97)	.75	1.21 (1.07-1.37)	.003
Cesarean delivery				
No	1 [Reference]	NA	1 [Reference]	NA
Yes	1.13 (0.01-1.42)	.28	1.04 (1.00-1.09)	.06
Maternal age at delivery, y				
<25	1 [Reference]	NA	1 [Reference]	NA
25-35	1.51 (0.53-4.26)	.44	1.24 (1.07-1.45)	.005
≥35	2.42 (0.90-6.52)	.08	1.43 (1.24-1.66)	<.001
Gestational diabetes				
No	1 [Reference]	NA	1 [Reference]	NA
Yes	1.41 (0.95-2.08)	.09	1.16 (1.07-1.25)	<.001
Gestational hypertension				
No	1 [Reference]	NA	1 [Reference]	NA
Yes	1.01 (0.47-2.17)	.98	1.16 (1.01-1.33)	.03
Socioeconomic status, quartile				
First (lowest)	1 [Reference]	NA	1 [Reference]	NA
Second	1.01 (0.74-1.39)	.93	1.05 (0.99-1.12)	.10
Third	1.12 (0.82-1.54)	.46	1.07 (1.00-1.13)	.04
Fourth (highest)	1.15 (0.84-1.58)	.40	1.03 (0.97-1.09)	.40
Residence				
Rural	1 [Reference]	NA	1 [Reference]	NA
Urban	1.03 (0.99-1.07)	.18	1.40 (1.12-1.75)	.003

Abbreviations: AHR, adjusted hazard ratio; NA, not applicable.

Low birth weight was significantly associated with an increased risk of CPP in both boys (AHR, 1.99; 95% CI, 1.21-3.26; *P* = .007) and girls (AHR, 1.17; 95% CI, 1.05-1.30; *P* = .003). In contrast, in girls only, significant associations with greater incidence of CPP were found for maternal age 25 to 35 years (AHR, 1.24; 95% CI, 1.07-1.45; *P* = .005) and 35 years or older (AHR, 1.43; 95% CI, 1.24-1.66; *P* < .001), gestational diabetes (AHR, 1.16; 95% CI, 1.07-1.25; *P* < .001), gestational hypertension (AHR, 1.16; 95% CI, 1.01-1.33; 1.01-1.33; *P* = .03), preterm birth (AHR, 1.26; 95% CI, 1.06-1.50), multiple births (AHR, 1.21; 95% CI, 1.07-1.37; *P* = .003), and residence in urban areas (AHR, 1.40; 95% CI, 1.12-1.75; *P* = .003). Of note, the associations between primary feeding type and CPP were slightly attenuated when BMI was included as a covariate (eTable 2 in Supplement 1). The subgroup analyses in children who were not born before term (eTable 3 in Supplement 1) or with low birth weight (eTable 4 in Supplement 1) yielded results consistent with those of the main analysis.

### Mediating Effects of Overweight and Obesity

Mediation analysis revealed that prepubertal overweight or obesity partially mediated the association between primary feeding type and CPP in both sexes (Table 3). Compared with exclusively breastfed children, formula-fed boys had a higher risk of CPP (total effect AHR, 1.20; bootstrap 95% CI, 1.15-1.25), with 7.2% (bootstrap 95% CI, 4.5%-12.1%) of the effect mediated through prepubertal adiposity. In girls, the total effect of formula feeding was greater (AHR, 1.59; bootstrap 95% CI, 1.50-1.67), with 17.8% (bootstrap 95% CI, 6.6%-30.0%) of the association mediated. Results were similar when prepubertal adiposity was treated as a continuous BMI variable (eTable 5 in Supplement 1).

**Table 3.** Mediation Effect of Prepubertal Overweight or Obesity on the Association Between Primary Feeding Type and Central Precocious Puberty

Variable	Male (n = 135 232)		Female (n = 187 499)	
	AHR (bootstrap 95% CI)^a^	*P* value	AHR (bootstrap 95% CI)^a^	*P* value
**Primary feeding type: formula-fed vs exclusively breastfed**				
Total effect	1.20 (1.15-1.25)	<.001	1.59 (1.50-1.67)	<.001
Controlled direct effect	1.17 (1.11-1.23)	<.001	1.66 (1.56-1.75)	<.001
Natural direct effect	1.18 (1.13-1.23)	<.001	1.48 (1.39-1.57)	<.001
Natural indirect effect	1.05 (1.05-1.06)	<.001	1.03 (1.03-1.04)	<.001
Percentage mediated	7.2 (4.5-12.1)	<.001	17.8 (6.6-30.0)	<.001
**Primary feeding type: mixed-fed vs exclusively breastfed**				
Total effect	1.17 (1.11-1.23)	<.001	1.44 (1.34-1.54)	<.001
Controlled direct effect	1.14 (1.07-1.21)	<.001	1.52 (1.40-1.63)	<.001
Natural direct effect	1.15 (1.09-1.21)	<.001	1.39 (1.29-1.49)	<.001
Natural indirect effect	1.06 (1.05-1.06)	<.001	1.04 (1.03-1.04)	<.001
Percentage mediated	9.3 (5.3-14.8)	<.001	12.4 (3.4-22.4)	<.001

Abbreviation: AHR, adjusted hazard ratio.

^a^ Models were adjusted for low birth weight, preterm birth, multiple birth, cesarean delivery, maternal age at delivery, gestational diabetes, gestational hypertension, socioeconomic status, and residence.

## Discussion

In this nationwide, retrospective cohort study, we found that children who were not exclusively breastfed during the first 4 to 6 months had a higher risk of CPP, regardless of sex. Overweight and obesity at prepubertal age were identified as potential mediators in the association between breastfeeding and CPP in both sexes.

In this study, breastfeeding during early infancy was associated with a lower likelihood of CPP, partially mediated by prepubertal overweight and obesity in both sexes. While the association between breastfeeding and pubertal development has been previously explored, studies that analyzed outcomes separately by sex remain limited. To date, 4 studies have included both boys and girls,^16,17,25,26^ with only 1 conducting a sex-stratified analysis.^16^ Kwok et al^25^ investigated the association between breastfeeding and childhood milk consumption and age at pubertal onset among Chinese children (boys and girls together) in Hong Kong and reported no significant association. Similarly, Karaolis-Danckert et al^17^ reported that children who were breastfed for more than 4 months experienced a delayed age at peak height velocity compared with children who were not breastfed; however, this association was attenuated and lost statistical significance after adjusting for covariates. In South Korea, Lee et al^26^ reported that breastfeeding for more than 6 months was protective against early pubertal development in both boys and girls; however, their analysis was not stratified by sex. In contrast, Hvidt et al^16^ examined boys and girls separately and found that boys who were not breastfed or had a shorter duration of breastfeeding experienced earlier pubertal onset than exclusively breastfed boys, while no significant association was observed for girls.

These differences in sex-specific associations may be attributed to ethnic differences. The cohort studied by Hvidt et al^16^ in Denmark comprised mostly White individuals, whereas our cohort included primarily East Asian individuals, mostly Korean. Evidence from other studies also supports ethnic differences in the association of feeding practices with pubertal development. In a multiethnic study, girls who were fed only formula presented early pubertal onset approximately 2.5 months earlier, but this was more pronounced among Black children and less pronounced among Asian or Pacific Islander children.^27^ There is evidence suggesting that breast development occurs earlier in Black girls,^28^ and the age at menarche also differs among Asian, Black, and White populations,^29,30,31^ indicating that the timing of hypothalamic-pituitary-gonadal axis activation may vary according to genetic or biological factors.

Differences in feeding practice patterns may also contribute to disparities in the association of breastfeeding with CPP across countries. In our study, the breastfeeding rate at 6 months was 46.0%, which was lower than the rates reported in Denmark (64.0%),^16^ the US (55.8%),^32^ and Sri Lanka (50.8%).^33^ Additionally, how breastmilk was given (either directly or via bottle feeding) or the duration of breastfeeding may also have influenced outcomes and were not included in our study design.

The risk of CPP was similarly elevated in both the mixed-fed and formula-fed groups, suggesting that the protective association may be specific to exclusive breastfeeding. In addition, mediation analysis suggested that prepubertal overweight or obesity possibly mediated the association between early-life breastfeeding and CPP. Several studies have explored the potential link between early-life nutrition and the timing of puberty, with childhood adiposity possibly serving as a key factor.^22,34,35^ Compared with formula-feeding, breastfeeding during the first year of life has been associated with a lower risk of childhood overweight or obesity.^36,37^ Additionally, girls with obesity may be more likely to develop CPP, although the association in boys remains unclear.^22^ Nonetheless, the proportion mediated was modest, suggesting that there are other biological pathways beyond prepubertal adiposity that may contribute to the association of breastfeeding and CPP.

One possible explanation for this finding is the association between insulin-like growth factor 1 (IGF-1) and sex hormone production. Although the concentration of IGF-1 in formula milk is lower than in human milk,^38^ formula-fed infants tend to have higher circulating IGF-1 concentrations at 3 months of age than breastfed infants.^39^ Elevated IGF-1 levels in infancy have been associated with increased linear growth^40^ and may accelerate pubertal development by enhancing sex steroid production and GnRH secretion.^41,42,43^

Another hypothesis involves differences in microbiome composition. Human milk contains beneficial bacteria that primarily shape the infant’s microbiota, influencing the gastrointestinal or immune system and neurologic development.^44^ Several studies have shown differences in the gut microbiome between breastfed and formula-fed infants. Breastfed infants present higher proportions of beneficial bacteria, such as *Bifidobacterium*, and lower microbial diversity, whereas formula-fed infants present greater diversity, with higher levels of Firmicutes and Proteobacteria.^45,46^ Furthermore, emerging evidence has linked gut microbiota composition to hormonal regulation, suggesting that the influence of breast milk on the gut may play a role in modifying the onset of puberty.^47,48^ Feeding patterns within the first 6 months of life may alter microbial equilibrium, potentially affecting childhood obesity and sex hormone secretion.^49^

Early infancy is a critical period of life for establishing key physiologic systems, including the gut microbiome and hormonal and metabolic pathways, that may influence pubertal timing.^50,51^ Future research is needed to elucidate the underlying biological mechanisms by which breastfeeding influences pubertal development. Furthermore, given the relatively low and declining rate of exclusive breastfeeding in South Korea (from 42.8% in 2010-2012 to 13.1% in 2019-2020),^52^ public health strategies to promote breastfeeding may be warranted. This downward trend may be attributable to factors such as increased maternal employment, limited parental leave policies, increased maternal age at delivery, and disparities in maternal educational levels and nutritional knowledge.^52,53^ To address these challenges, nationwide educational campaigns and the development of a breastfeeding-friendly workplace environment may help support sustained breastfeeding practices.

### Strengths and Limitations

To our knowledge, this study is the first to investigate the mediating role of prepubertal adiposity in the association between early-life feeding type and CPP in a large population-based cohort, providing novel insights into the mechanisms linking early-life nutrition and pubertal timing. This study benefits from the use of a nationwide cohort with long-term follow-up from infancy to childhood and comprehensive adjustment for key confounders, including socioeconomic status, perinatal characteristics, and maternal health conditions. Furthermore, as we used register-based data with strict diagnostic criteria for CPP and a clear chronologic sequence of exposure, mediator, and outcome, we minimized misclassification bias and satisfied the counterfactual requirement of temporality for mediation analysis. Finally, sex-stratified and sensitivity analyses confirmed the robustness of our study findings.

This study also had several limitations. First, data on maternal age at menarche, which may influence the timing of puberty onset in daughters, were not available because of the limitations of the NHIS database. Second, only children treated with GnRH agonists under the insurance reimbursement system were included in the CPP cases. The Korean NHIS uses a fee-for-service payment model, which may lead to potential overdiagnosis of CPP to meet the reimbursement criteria. However, clinical confirmation of CPP based on established diagnostic criteria may reduce the potential impact of this concern. Third, we could not assess the dose-dependent association between breastfeeding duration and CPP risk. Fourth, the manner in which the children were given breast milk (bottle vs direct feeding from the breast, which indicates the time of skin-to-skin contact between the mother and infant), was not included in the analysis. Furthermore, overweight or obesity at age 66 to 71 months was modeled as a fixed covariate rather than a time-varying variable due to data structure, which may have limited the ability to fully reflect its temporal variability. Finally, given the observational design of the study, causality cannot be inferred due to the potential for unmeasured or residual confounding.

## Conclusions

In this retrospective cohort study, exclusive breastfeeding during early infancy was associated with a lower risk of CPP in both boys and girls, and this association was partially mediated by prepubertal overweight or obesity. Further longitudinal prospective studies are needed to evaluate the association of breastfeeding with pubertal timing and progression.

## Notes

Supplement 1.
eMethods.

eReferences.
**eTable 1.** Detailed Information on *ICD-10* Diagnostic Codes Used in the Study **eTable 2.** Associations Between Primary Feeding Type and Central Precocious Puberty With Additional Adjustment for Prepubertal Adiposity **eTable 3.** Associations Between Primary Feeding Type and Central Precocious Puberty in Children Without Preterm Birth **eTable 4.** Associations Between Primary Feeding Type and Central Precocious Puberty in Children Without Low Birth Weight **eTable 5.** Mediation Effect of Prepubertal Body Mass Index on the Association Between Primary Feeding Type and Central Precocious Puberty **eFigure 1.** Directed Acyclic Graph Showing Association Between Primary Feeding Type and Central Precocious Puberty **eFigure 2.** Kaplan-Meier Curves of Central Precocious Puberty–Free Survival Stratified by Primary Feeding Type in Boys and Girls

Supplement 2.
Data Sharing Statement
