# Supplementary material for: Breastfeeding, Prepubertal Adiposity, and Development of Precocious Puberty
Source: JAMA Netw Open. 2025 Aug 18;8(8):e2527455. doi: 10.1001/jamanetworkopen.2025.27455 (PMC12362225; doi:10.1001/jamanetworkopen.2025.27455)
Supplement: Supplement 1. — eMethods. eReferences. eTable 1. Detailed Information on ICD-10 Diagnostic Codes Used in the Study eTable 2. Associations Between Primary Feeding Type and Central Precocious Puberty With Additional Adjustment for Prepubertal Adiposity eTable 3. Associations Between Primary Feeding Type and Central Precocious Puberty in Children Without Preterm Birth eTable 4. Associations Between Primary Feeding Type and Central Precocious Puberty in Children Without Low Birth Weight eTable 5. Mediation Effect of Prepubertal Body Mass Index on the Association Between Primary Feeding Type and Central Precocious Puberty eFigure 1. Directed Acyclic Graph Showing Association Between Primary Feeding Type and Central Precocious Puberty eFigure 2. Kaplan-Meier Curves of Central Precocious Puberty–Free Survival Stratified by Primary Feeding Type in Boys and Girls [file jamanetwopen-e2527455-s001.pdf]

Supplemental Online Content

Choe Y, Ryu S, Choi J, et al. Breastfeeding, prepubertal adiposity, and development of precocious puberty. *JAMA Netw Open*. 2025;8(8):e2527455. doi:10.1001/jamanetworkopen.2025.27455

eMethods.

eReferences.

eTable 1. STROBE Statement—Checklist of Items That Should Be Included in Reports of Cohort Studies

eTable 2. Detailed Information on *ICD-10* Diagnostic Codes Used in the Study

eTable 3. Associations Between Primary Feeding Type and Central Precocious PubertyWith Additional Adjustment for Prepubertal Adiposity

eTable 4. Associations Between Primary Feeding Type and Central Precocious Puberty in Children Without Preterm Birth

eTable 5. Associations Between Primary Feeding Type and Central Precocious Puberty in Children Without Low Birth Weight

eTable 6. Mediation Effect of Prepubertal Body Mass Index on the Association Between Primary Feeding Type and Central Precocious Puberty

eFigure 1. Directed Acyclic Graph Showing Association Between Primary Feeding Type and Central Precocious Puberty

eFigure 2. Kaplan-Meier Curves of Central Precocious Puberty–Free Survival Stratified by Primary Feeding Type in Boys and Girls

This supplemental material has been provided by the authors to give readers additional information about their work.

© 2025 Choe Y et al. *JAMA Network Open*.

eMethods

Statistical Analysis

Demographic variables and group comparisons

Data are expressed as the mean ± SD for continuous variables or the number (percentage) for categorical variables. To compare the three groups based on feeding type (exclusively breastfed, formula-fed, and mixed-fed), we conducted one-way analysis of variance (ANOVA) for continuous variables, and the chi-square test for categorical variables.

Cox proportional hazard model

Factors affecting CPP were analyzed using a multivariable Cox proportional hazards model adjusted for confounders, and the results are presented as adjusted hazard ratios (aHRs) and 95% CIs. The proportional hazards assumption was evaluated using the scaled Schoenfeld residuals test, as proposed by Grambsch and Therneau[1], and by visual inspection of residual plots. The result results showed that all covariates and global model yielded P vales > .05. The residual plots did not show any specific pattern or trend over time, suggesting that the assumption was adequately satisfied for all variables. In addition, Kaplan-Meier curves were plotted to estimate CPP-free survival by primary feeding type, and statistical differences between groups were evaluated using the log-rank test.

Sensitivity analyses were performed to confirm the robustness of the findings. First, the analysis was repeated with additional adjustment for overweight/obesity during the prepubertal period, which had been excluded from the main model due to its potential role for mediator. Second, children born preterm (N=6 636) were excluded. Third, children with low birth weight (<2.5 kg) were excluded (N=14 656). The second and third analyses were performed to minimize the potential influence of perinatal conditions on pubertal onset.[2] In the mediation analysis, we conducted a sensitivity analysis using prepubertal BMI as the mediator, modelled as a continuous variable.

Mediation analysis

To examine whether prepubertal adiposity (overweight or obesity) mediates the association between primary feeding type and CPP, we conducted a counterfactual, regression-based mediation analysis described by Vanderweele.[3,4] This method accounts for potential exposure-mediator interaction and allows for appropriate adjustment of mediator-outcome confounders. Covariates included in the models were selected *a priori* based on clinical relevance and a directed acyclic graph framework: preterm birth, low birth weight, multiple birth, caesarean delivery, maternal age at delivery, gestational diabetes mellitus, gestational hypertension, socioeconomic status, and residence. We performed a stepwise approach as follows:

Step 1. Exposure – Mediator Model: Multivariable logistic regression of prepubertal overweight or obesity (dichotomous variable) on primary feeding type and covariates.

Step 2. Mediator – Outcome Model: Cox proportional hazard regression of CPP on primary feeding type, prepubertal adiposity, and covariates.

Step 3. Sensitivity analysis: Linear regression analysis was conducted in step 1 when prepubertal adiposity is treated as continuous BMI variable.

The following describes the decomposition of the total effect into distinct components in counterfactual-based mediation analysis.[5,6] The total effect represents the overall change in the risk of CPP if the exposure were changed from breastmilk feeding to either formula feeding or mixed feeding. The controlled direct effect represents the overall change in the risk of CPP when the exposure was changed from breastmilk feeding to formula feeding or mixed feeding while the mediator status (childhood overweight or obesity) was fixed at a specific level for all individuals. The natural direct effect represents the portion of the total effect not explained by prepubertal adiposity. The natural indirect effect represents the portion of the total effect mediated through prepubertal adiposity. The percentage mediated indicates the proportion of the total effect attributable to the indirect (mediated) pathway. All statistical analyses were conducted using SAS Enterprise Guide version 7.1 (SAS Institute Inc., Cary, NC, USA) and R 4.4.0 (R Foundation for Statistical Computing, Vienna, Austria). Mediation analysis was performed with the SAS PROC CAUSALMED. We derived estimates and 95% CIs for natural direct, natural indirect, and total effects via 5 000 bootstrap iterations. $P < .05$ was considered statistically significant, and all hypothesis tests were 2-sided.

© 2025 Choe Y et al. *JAMA Network Open*.

eReferenes

1. Grambsch PM, Therneau TM. Proportional hazards tests and diagnostics based on weighted residuals. *Biometrika*. 1994;81(3):515-515.
2. Ibáñez L, Ferrer A, Marcos MV, Hierro FR, de Zegher F. Early puberty: rapid progression and reduced final height in girls with low birth weight. *Pediatrics*. 2000;106(5):E72. doi:10.1542/peds.106.5.e72
3. VanderWeele TJ. *Explanation in Causal Inference: Methods for Mediation and Interaction.* Oxford University Press; 2015:20-46.
4. VanderWeele TJ. Mediation analysis. In: Lash TL, VanderWeele TJ, Haneuse S, Rothman KJ, eds. *Modern Epidemiology*. 4th ed. Wolters Kluwer; 2021:973-996:chap 27.
5. VanderWeele TJ. A Unification of Mediation and Interaction: A 4-Way Decomposition. *Epidemiology*. 2014;25(5):749-761. doi:10.1097/ede.0000000000000121
6. Li Y, Yoshida K, Kaufman JS, Mathur MB. A brief primer on conducting regression-based causal mediation analysis. *Psychol Trauma*. Sep 2023;15(6):930-938. doi:10.1037/tra0001421

© 2025 Choe Y et al. *JAMA Network Open*.

eTable 1. STROBE Statement—Checklist of Items That Should Be Included in Reports of Cohort Studies

	Item No	Recommendation	pages
**Title and abstract**	1	(*a*) Indicate the study's design with a commonly used term in the title or the abstract	1-5
		(*b*) Provide in the abstract an informative and balanced summary of what was done and what was found	1-5
**Introduction**			
Background/rationale	2	Explain the scientific background and rationale for the investigation being reported	6-7
Objectives	3	State specific objectives, including any prespecified hypotheses	6-7
**Methods**			
Study design	4	Present key elements of study design early in the paper	7-10
Setting	5	Describe the setting, locations, and relevant dates, including periods of recruitment, exposure, follow-up, and data collection	7-10
Participants	6	(*a*) Give the eligibility criteria, and the sources and methods of selection of participants. Describe methods of follow-up	7-10
		(*b*) For matched studies, give matching criteria and number of exposed and unexposed	7-10
Variables	7	Clearly define all outcomes, exposures, predictors, potential confounders, and effect modifiers. Give diagnostic criteria, if applicable	7-10
Data sources/ measurement	8*	For each variable of interest, give sources of data and details of methods of assessment (measurement). Describe comparability of assessment methods if there is more than one group	7-10
Bias	9	Describe any efforts to address potential sources of bias	7-10
Study size	10	Explain how the study size was arrived at	7-10
Quantitative variables	11	Explain how quantitative variables were handled in the analyses. If applicable, describe which groupings were chosen and why	7-10
Statistical methods	12	(*a*) Describe all statistical methods, including those used to control for confounding	7-10
		(*b*) Describe any methods used to examine subgroups and interactions	7-10
		(*c*) Explain how missing data were addressed	7-10, Fig. 1
		(*d*) If applicable, explain how loss to follow-up was addressed	7-10, Fig. 1
		(*e*) Describe any sensitivity analyses	7-10
**Results**			
Participants	13*	(a) Report numbers of individuals at each stage of study—eg numbers potentially eligible, examined for eligibility, confirmed eligible, included in the study, completing follow-up, and analysed	7-8
		(b) Give reasons for non-participation at each stage	7-8
		(c) Consider use of a flow diagram	Fig. 1
Descriptive data	14*	(a) Give characteristics of study participants (eg demographic, clinical, social) and information on exposures and potential confounders	8-10
		(b) Indicate number of participants with missing data for each variable of interest	7-8
		(c) Summarise follow-up time (eg, average and total amount)	7-8
Outcome data	15*	Report numbers of outcome events or summary measures over time	10-12
Main results	16	(*a*) Give unadjusted estimates and, if applicable, confounder-adjusted estimates and their precision (eg, 95% confidence	10-12

© 2025 Choe Y et al. *JAMA Network Open*.

		interval). Make clear which confounders were adjusted for and why they were included	
		(*b*) Report category boundaries when continuous variables were categorized	10-12
		(*c*) If relevant, consider translating estimates of relative risk into absolute risk for a meaningful time period	10-12
Other analyses	17	Report other analyses done—eg analyses of subgroups and interactions, and sensitivity analyses	10-12
**Discussion**			
Key results	18	Summarise key results with reference to study objectives	12
Limitations	19	Discuss limitations of the study, taking into account sources of potential bias or imprecision. Discuss both direction and magnitude of any potential bias	16
Interpretation	20	Give a cautious overall interpretation of results considering objectives, limitations, multiplicity of analyses, results from similar studies, and other relevant evidence	12-16
Generalisability	21	Discuss the generalisability (external validity) of the study results	12-16
**Other information**			
Funding	22	Give the source of funding and the role of the funders for the present study and, if applicable, for the original study on which the present article is based	17

*Give information separately for exposed and unexposed groups.

Note: An Explanation and Elaboration article discusses each checklist item and gives methodological background and published examples of transparent reporting. The STROBE checklist is best used in conjunction with this article (freely available on the Web sites of PLoS Medicine at http://www.plosmedicine.org/, Annals of Internal Medicine at http://www.annals.org/, and Epidemiology at http://www.epidem.com/). Information on the STROBE Initiative is available at http://www.strobe-statement.org.

© 2025 Choe Y et al. *JAMA Network Open*.

eTable 2. Detailed Information on *ICD-10* Diagnostic Codes Used in the Study

Definition	ICD-10 codes
Central precocious puberty	E22.8, E30.1, E30.8
Congenital anomaly	Q90-Q99
Malignant neoplasm	C00-C96
Neurologic disorder	G00-G99
Endocrinologic disorder	E20-E35
Chronic kidney disease	N18
Preterm birth	P07
Gestational diabetes	E08-E13, O24
Gestational hypertension	O13
Cesarean delivery	O82

© 2025 Choe Y et al. *JAMA Network Open*.

eTable 3. Associations Between Primary Feeding Type and Central Precocious Puberty With Additional Adjustment for Prepubertal Adiposity

	Male (n=135 232)		Female (n=187 499)	
	AHR (95% CI)	*P* value	AHR (95% CI)	*P* value
Primary feeding type				
Exclusively breastfed	1 [Reference]	NA	1 [Reference]	NA
Formula-fed	1.15 (1.10-1.21)	<.001	1.58 (1.23-2.05)	<.001
Mixed-fed	1.13 (1.07-1.20)	<.001	1.44 (1.06-1.95)	<.001
Low birth weight				
No	1 [Reference]	NA	1 [Reference]	NA
Yes	2.07 (1.27-3.39)	.004	1.19 (1.07-1.32)	<.001
Preterm birth				
No	1 [Reference]	NA	1 [Reference]	NA
Yes	1.47 (0.68-3.21)	.33	1.28 (1.07-1.51)	.01
Multiple birth				
No	1 [Reference]	NA	1 [Reference]	NA
Yes	1.06 (0.59-1.90)	.84	1.19 (1.06-1.35)	.01
Cesarean delivery				
No	1 [Reference]	NA	1 [Reference]	NA
Yes	1.10 (0.88-1.39)	.41	1.03 (0.98-1.07)	.21
Maternal age at delivery, y				
<25	1 [Reference]	NA	1 [Reference]	NA
25–35	1.55 (0.55-4.38)	.41	1.25 (1.07-1.46)	.004
≥35	2.50 (0.93-6.71)	.07	1.44 (1.25-1.67)	<.001
Gestational diabetes				
No	1 [Reference]	NA	1 [Reference]	NA
Yes	1.38 (0.93-2.03)	.11	1.15 (1.06-1.24)	<.001
Gestational hypertension				
No	1 [Reference]	NA	1 [Reference]	NA
Yes	0.97 (0.45-2.08)	.94	1.14 (0.99-1.31)	.06
Socioeconomic status, quartile				
1st (Lowest)	1 [Reference]	NA	1 [Reference]	NA
2nd	1.03 (0.75-1.41)	.88	1.06 (1.00-1.12)	.07
3rd	1.15 (0.84-1.57)	.39	1.07 (1.01-1.14)	.02
4th (Highest)	1.18 (0.86-1.63)	.30	1.04 (0.98-1.10)	.21
Residence				
Rural	1 [Reference]	NA	1 [Reference]	NA
Urban	1.03 (0.99-1.08)	.11	1.41 (1.13-1.76)	.003
Overweight/obesity[a]				
No	1 [Reference]	NA	1 [Reference]	NA
Yes	1.45 (1.38-1.52)	<.001	2.08 (1.63-2.65)	<.001

CPP, central precocious puberty; HR, hazard ratio; CI, confidence interval
[a]From the seventh infant health checkup (conducted at age 66–71 months)

© 2025 Choe Y et al. *JAMA Network Open*.

eTable 4. Associations Between Primary Feeding Type and Central Precocious Puberty in Children Without Preterm Birth

	Male (n=132 328)		Female (n=183 767)	
	AHR (95% CI)	*P* value	AHR (95% CI)	*P* value
Primary feeding type				
Exclusively breastfed	1 [Reference]	NA	1 [Reference]	NA
Formula-fed	1.16 (1.11-1.21)	<.001	1.62 (1.25-2.10)	<.001
Mixed-fed	1.13 (1.07-1.20)	<.001	1.40 (1.03-1.91)	<.001
Low birth weight				
No	1 [Reference]	NA	1 [Reference]	NA
Yes	1.96 (1.15-3.33)	.01	1.21 (1.08-1.34)	<.001
Multiple birth				
No	1 [Reference]	NA	1 [Reference]	NA
Yes	1.27 (0.65-2.47)	.49	1.22 (1.07-1.39)	.003
Cesarean delivery				
No	1 [Reference]	NA	1 [Reference]	NA
Yes	1.12 (0.89-1.41)	.35	1.04 (1.00-1.09)	.08
Maternal age at delivery, y				
<25	1 [Reference]	NA	1 [Reference]	NA
25–35	1.59 (0.54-4.36)	.42	1.25 (1.07-1.45)	.01
≥35	2.37 (0.88-6.38)	.09	1.44 (1.24-1.66)	<.001
Gestational diabetes				
No	1 [Reference]	NA	1 [Reference]	NA
Yes	1.42 (0.95-2.11)	.09	1.16 (1.07-1.25)	<.001
Gestational hypertension				
No	1 [Reference]	NA	1 [Reference]	NA
Yes	0.89 (0.36-2.16)	.79	1.11 (0.95-1.28)	.19
Socioeconomic status, quartile				
1st (Lowest)	1 [Reference]	NA	1 [Reference]	NA
2nd	0.99 (0.72-1.36)	.93	1.05 (0.99-1.11)	.14
3rd	1.10 (0.80-1.51)	.55	1.06 (1.00-1.12)	.06
4th (Highest)	1.15 (0.83-1.58)	.40	1.02 (0.96-1.08)	.63
Residence				
Rural	1 [Reference]	NA	1 [Reference]	NA
Urban	1.03 (0.99-1.07)	.18	1.43 (1.14-1.79)	.002

AHR, hazard ratio; NA, not applicable.

© 2025 Choe Y et al. *JAMA Network Open*.

eTable 5. Associations Between Primary Feeding Type and Central Precocious Puberty in children Without Low Birth Weight

	Male (n=129 878)		Female (n=178 197)	
	AHR (95% CI)	*P* value	AHR (95% CI)	*P* value
Primary feeding type				
Exclusively breastfed	1 [Reference]	NA	1 [Reference]	NA
Formula-fed	1.17 (1.11-1.23)	<.001	1.56 (1.20-2.03)	<.001
Mixed-fed	1.13 (1.07-1.20)	<.001	1.40 (1.03-1.92)	<.001
Preterm birth				
No	1 [Reference]	NA	1 [Reference]	NA
Yes	2.69 (0.38-19.27)	.32	1.04 (0.80-1.37)	.77
Multiple birth				
No	1 [Reference]	NA	1 [Reference]	NA
Yes	0.95 (0.47-1.93)	.88	1.15 (0.99-1.33)	.07
Cesarean delivery				
No	1 [Reference]	NA	1 [Reference]	NA
Yes	1.14 (0.90-1.45)	.27	1.05 (1.00-1.10)	.03
Maternal age at delivery, y				
<25	1 [Reference]	NA	1 [Reference]	NA
25–35	1.45 (0.51-4.13)	.48	1.23 (1.05-1.44)	.01
≥35	2.25 (0.84-6.07)	.11	1.42 (1.23-1.64)	<.001
Gestational diabetes				
No	1 [Reference]	NA	1 [Reference]	NA
Yes	1.38 (0.92-2.09)	.13	1.17 (1.08-1.26)	<.001
Gestational hypertension				
No	1 [Reference]	NA	1 [Reference]	NA
Yes	0.66 (0.21-2.07)	.48	1.07 (0.91-1.26)	.41
Socioeconomic status, quartile				
1st (Lowest)	1 [Reference]	NA	1 [Reference]	NA
2nd	0.98 (0.71-1.37)	.92	1.06 (1.00-1.12)	.08
3rd	1.07 (0.77-1.49)	.67	1.06 (1.00-1.13)	.06
4th (Highest)	1.20 (0.86-1.66)	.28	1.02 (0.96-1.08)	.53
Residence				
Rural	1 [Reference]	NA	1 [Reference]	NA
Urban	1.03 (0.99-1.07)	.20	1.44 (1.13-1.80)	.003

AHR, hazard ratio; NA, not applicable.

© 2025 Choe Y et al. *JAMA Network Open*.

eTable 6. Mediation Effect of Prepubertal Body Mass Index on the Association Between Primary Feeding Type and Central Precocious Puberty

	Male (n=135 232)		Female (n=187 499)	
	AHR (Bootstrap 95% CI)	*P* value	AHR (Bootstrap 95% CI)	*P* value
Primary feeding type: formula-fed vs. exclusively breastfed				
Total effect	1.19 (1.14–1.24)	<.001	1.57 (1.48–1.65)	<.001
Controlled direct effect	1.19 (1.14–1.24)	<.001	1.66 (1.57–1.74)	<.001
Natural direct effect	1.18 (1.13–1.23)	<.001	1.46 (1.38–1.55)	<.001
Natural indirect effect	1.05 (1.05–1.05)	<.001	1.03 (1.03–1.03)	<.001
Percentage mediated	6.62 (2.00–13.03)	<.001	18.19 (4.65–33.50)	<.001
Primary feeding type: mixed-fed vs. exclusively breastfed				
Total effect	1.17 (1.11–1.23)	<.001	1.44 (1.34–1.54)	<.001
Controlled direct effect	1.16 (1.10–1.22)	<.001	1.53 (1.43–1.63)	<.001
Natural direct effect	1.15 (1.09–1.21)	<.001	1.38 (1.28–1.48)	<.001
Natural indirect effect	1.05 (1.05–1.06)	<.001	1.03 (1.03–1.04)	<.001
Percentage mediated	8.88 (2.23–15.23)	<.001	12.22 (4.67–20.63)	<.001

Models were adjusted for preterm birth, low birth weight, multiple birth, cesarean delivery, maternal age at delivery, gestational diabetes, gestational hypertension, socioeconomic status, and residence.
AHR, adjusted hazard ratio.

© 2025 Choe Y et al. *JAMA Network Open*.

eFigure 1. Directed Acyclic Graph Showing Association Between Primary Feeding Type, Prepubertal Overweight or Obesity, and Central Precocious Puberty

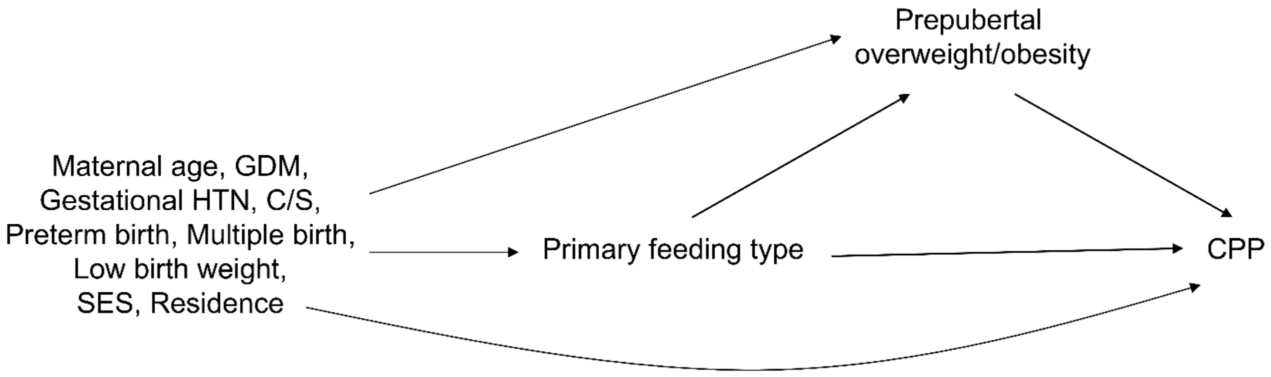

The minimal sufficient adjustment set was identified based on this model.
CPP, central precocious puberty; SES, socioeconomic status; C/S, cesarean delivery; GDM, gestational diabetes mellitus; HTN, hypertension.

© 2025 Choe Y et al. *JAMA Network Open*.

eFigure 2. Kaplan-Meier Curves of Central Precocious Puberty–Free Survival Stratified by Primary Feeding Type in Boys and Girls

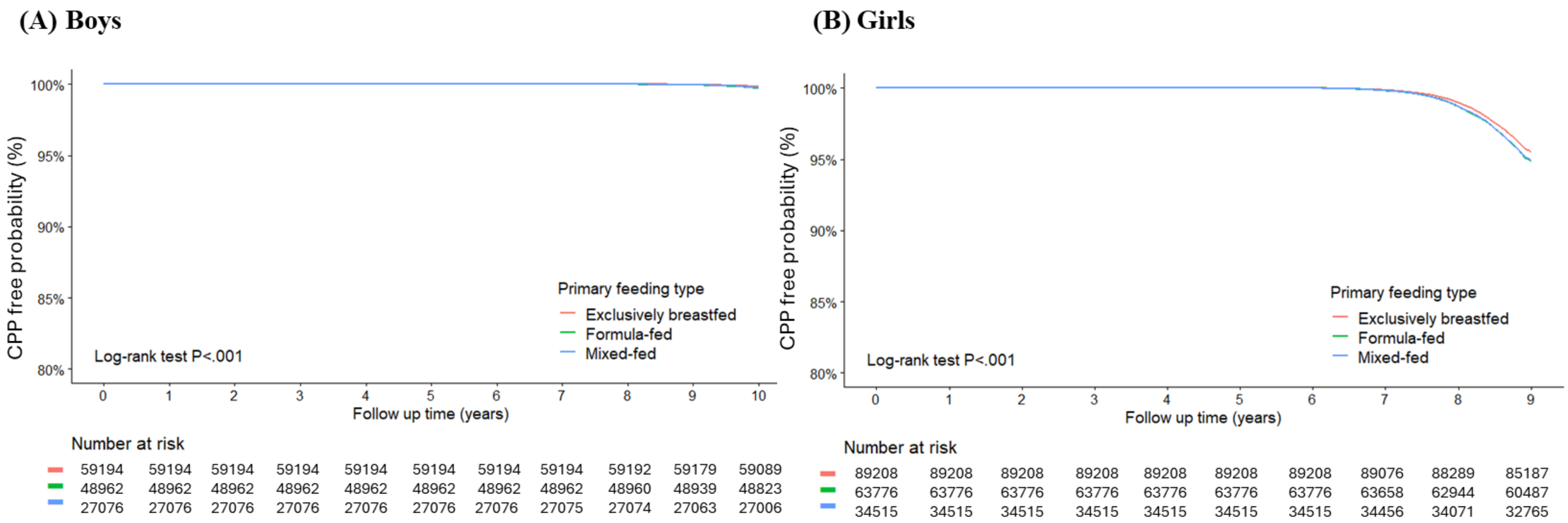

CPP, central precocious puberty.

© 2025 Choe Y et al. *JAMA Network Open*.
