# Supplementary material for: Breastfeeding, Prepubertal Adiposity, and Development of Precocious Puberty
Source: JAMA Netw Open. 2025 Aug 18;8(8):e2527455. doi: 10.1001/jamanetworkopen.2025.27455 (PMC12362225; doi:10.1001/jamanetworkopen.2025.27455)
Supplement: Supplement 2. — Data Sharing Statement [file jamanetwopen-e2527455-s002.pdf]

Data Sharing Statement

Choe. Breastfeeding, Prepubertal Adiposity, and Development of Precocious Puberty. *JAMA Netw Open*. Published August 18, 2025. doi:10.1001/jamanetworkopen.2025.27455

Data
Data available: No

Additional Information
Explanation for why data not available: The data that support the findings of this study are available from South Korean National Health Insurance Service Database, but restrictions apply to the availability of these data, which were used under license for the current study and therefore are not publicly available.
